# Supplementary material for: Using dynamic microsimulation to project cognitive function in the elderly population
Source: PLoS One. 2022 Sep 15;17(9):e0274417. doi: 10.1371/journal.pone.0274417 (PMC9477290; doi:10.1371/journal.pone.0274417)
Supplement: S1 Appendix — (DOCX) [file pone.0274417.s001.docx]

**Technical Appendix**

Table A-1. Proxy interview cognitive impairment rating from Health and Retirement Study, 2006-2016.

|  | 2006 | 2008 | 2010 | 2012 | 2014 | 2016 |
| --- | --- | --- | --- | --- | --- | --- |
| No reason to think the respondent has any cognitive limitations | 608 | 522 | 636 | 480 | 429 | 394 |
| The respondent may have some cognitive limitations but could probably do the interview | 92 | 90 | 91 | 81 | 73 | 58 |
| The respondent has cognitive limitations that prevent him/her from being interviewed | 560 | 528 | 655 | 584 | 547 | 489 |

Table A-2. TICS27 and mortality transition model.

| Variables | TICS27 Coefficient  (Std. Err) | Mortality Coefficient (Std.Err) |
| --- | --- | --- |
| Main effects |  |  |
| Two-year lag TICS score | 0.092 (0.001)*** |  |
| Four-year lag TICS score | 0.096 (0.002)*** |  |
| Non-Hispanic Black | -0.253 (0.014)*** | 0.026 (0.036) |
| Hispanic | -0.169 (0.019)*** | -0.072 (0.050) |
| Did not graduate high school | -0.155 (0.016)*** | -0.071 (0.033)* |
| At least some college | 0.197 (0.009)*** | -0.089 (0.029)** |
| Male | -0.109 (0.010)*** | -0.419 (0.901) |
| Slope of age spline before age 65 | -0.001 (0.001) | 0.019 (0.010) |
| Slope of age spline ages 65-74 | -0.017 (0.001)*** | 0.031 (0.006)*** |
| Slope of age spline ages 75 and older | -0.035 (0.001)*** |  |
| Slope of age spline ages 75-84 |  | 0.045 (0.005)*** |
| Slope of age spline ages 85 and older |  | 0.056 (0.005)*** |
| Ever diagnosed with heart problems | -0.013 (0.011) | 0.157 (0.032)*** |
| Ever diagnosed with stroke | 0.129 (0.016)*** | 0.166 (0.035)*** |
| Ever diagnosed with cancer | 0.001 (0.012) | 0.372 (0.030)*** |
| Ever diagnosed with hypertension | -0.036 (0.009)*** | 0.094 (0.030)** |
| Ever diagnosed with diabetes | -0.069 (0.010)*** | 0.156 (0.031)*** |
| Ever diagnosed with lung disease | -0.043 (0.015)** | 0.278 (0.035)*** |
| Heart attack in past 2 years | -0.075 (0.033)* | -0.108 (0.081) |
| Working for pay | 0.094 (0.010)*** |  |
| Widowed | -0.030 (0.012)* | 0.039 (0.028) |
| Ever smoked | -0.043 (0.008)*** |  |
| Verified ADOD/MCI ever | -0.710 (0.015)*** |  |
| Delta age | -0.137 (0.021)*** |  |
| Lag log BMI below 30 | 0.131 (0.035)*** |  |
| Lag log BMI above 30 | -0.009 (0.044) |  |
| Difficulty with one IADL |  | 0.190 (0.043)*** |
| Difficulty with two or more IADLs |  | 0.534 (0.044)*** |
| Difficulty with one ADL |  | 0.222 (0.038)*** |
| Difficulty with two ADLSs |  | 0.363 (0.049)*** |
| Difficulty with three or more ADLs |  | 0.640 (0.040)*** |
| Current smoker |  | 0.179 (0.046)*** |
| Diagnosed with heart problems by age 50 |  | -0.002 (0.113) |
| Diagnosed with stroke by age 50 |  | 0.036 (0.260) |
| Diagnosed with cancer by age 50 |  | -0.038 (0.063) |
| Diagnosed with hypertension by age 50 |  | -0.046 (0.063) |
| Diagnosed with diabetes by age 50 |  | 0.097 (0.055) |
| Diagnosed with lung disease by age 50 |  | -0.138 (0.180) |
| Ever smoked at age 50 |  | 0.083 (0.033) |
| Current smoker at age 50 |  | 0.183 (0.040)*** |
| Ever diagnosed with congestive heart failure |  | 0.290 (0.044)*** |
| Constant |  | -3.755 (0.590)*** |
| Gender interaction |  |  |
| Male and did not graduate high school | 0.046 (0.025) | 0.132 (0.050)** |
| Male and non-Hispanic Black | 0.046 (0.023)* | -0.017 (0.056) |
| Male and Hispanic | 0.046 (0.027) | -0.010 (0.075) |
| Male and at least some college |  | 0.035 (0.043) |
| Change in slope of age spline for males before age 65 |  | 0.008 (0.015) |
| Change in slope of age spline for males ages 65-74 |  | 0.004 (0.008) |
| Change in slope of age spline for males ages 75-84 |  | -0.006 (0.007) |
| Change in slope of age spline for males age 85 and older |  | 0.031 (0.008)*** |
| Male and ever diagnosed with congestive heart failure |  | -0.024 (0.063) |
| Male and ever diagnosed with heart problem |  | -0.051 (0.045) |
| Male and ever diagnosed with stroke |  | -0.097 (0.052) |
| Male and ever diagnosed with cancer |  | -0.084 (0.043) |
| Male and ever diagnosed with hypertension |  | 0.025 (0.043) |
| Male and ever diagnosed with diabetes |  | -0.013 (0.044) |
| Male and ever diagnosed with lung disease |  | 0.095 (0.051) |
| Male and heart attack within past 2 years |  | 0.198 (0.109) |
| Male and difficulty with one IADL |  | -0.081 (0.065) |
| Male and difficulty with two or more IADLs |  | -0.030 (0.072) |
| Male and difficulty with one ADL |  | 0.049 (0.058) |
| Male and difficulty with two ADLs |  | 0.025 (0.076) |
| Male and difficulty with three or more ADLs |  | 0.034 (0.065) |
| Male and current smoker |  | 0.103 (0.065) |
| Male and widowed |  | 0.027 (0.049) |
| Male and diagnosed with heart problems by age 50 |  | 0.080 (0.148) |
| Male and diagnosed with stroke by age 50 |  | -0.218 (0.448) |
| Male and diagnosed with cancer by age 50 |  | -0.143 (0.146) |
| Male and diagnosed with hypertension by age 50 |  | 0.199 (0.108) |
| Male and diagnosed with diabetes by age 50 |  | 0.070 (0.084) |
| Male and diagnosed with lung disease at age 50 |  | -0.340 (0.399) |
| Male and ever smoked by age 50 |  | -0.031 (0.049) |
| Male and current smoker at age 50 |  | -0.006 (0.053) |

Notes: *, significant at α=0.05; **, significant at α=0.01; ***, significant at α=0.001.
