# Supplementary material for: Using dynamic microsimulation to project cognitive function in the elderly population
Source: PLoS One. 2022 Sep 15;17(9):e0274417. doi: 10.1371/journal.pone.0274417 (PMC9477290; doi:10.1371/journal.pone.0274417)
Supplement: Attachment — Submitted filename: response_to_reviewers.docx [file pone.0274417.s002.docx]

**Point-by-point response to the editor and reviewers**

We thank the editors and reviewers for their time and attention in improving this manuscript. The comments strengthen the manuscript and add clarity, and we appreciate the opportunity to address these issues. To summarize the changes to the manuscript, we have copied the editor’s and reviewers’ comments below, verbatim in **bold**, along with our responses in regular text, and quotes from the manuscript in *italics*.

**Response to Editor**

1. **Kindly enhance the discussion part by elaborating how dynamic microsimulation could enhance the cognitive function in elderly people.**

We thank the reviewer for this excellent suggestion. We added the following section in the Discussion to demonstrate more clearly FEM’s role in enhancing cognitive function in elderly population. We highlighted its usefulness in demonstrating value of treatment and identification of people at risk of cognitive impairment, which are two important components of potentially enhancing cognitive function in the elderly population.

*With Aduhelm approved as the first ADOD DMT and more DMTs in the development pipeline, the future looks promising. Though crucial, availability of DMT is only one step in enhancing cognitive function in elderly population. Demonstrating value of treatment and identification of people at risk of cognitive impairment are two very important components as well. FEM microsimulation could help with these. Understanding the long-term impact of ADOD DMTs beyond direct medical expenditure is crucial to its value demonstration. [39]*

*[39]	Barbarino PG, Anders; Neumann Peter J. Long-term value demonstration in Alzheimer’s disease: evidence needs. Signal. 2022; (07).*

2. **Future directions is needed for better research projection.**

We would like to thank the reviewer for pointing out this omission. We edited the Discussion section, to include future research directions. We outline two approaches: 1) improving FEM TICS27’s performance by utilizing additional predictors; 2) applying FEM TICS27 to different contexts and countries to analyze differences in cognitive trajectory:

*Future development of FEM TICS27 with genotype, blood-based biomarker variables, behavioral symptoms and history of medication, which are available for a subsample of the HRS, will possibly improve its performance for people with MCI. Additionally, future applications of FEM TICS27 will include analyses of differences in cognitive trajectory by education, initial cognitive status, and race and ethnicity. The model can also be implemented in microsimulations for other countries.*

**Response to Reviewer 1**

1. **This study is of great significance and has high clinical value. The FEM TICS27 model demonstrates its predictive accuracy for both two- and ten-year cognitive outcomes. I would like to know what the prediction results of other years are by this model? Thank you!**

We appreciate the reviewer’s acknowledgment of our study’s significance. And we agree that it is important to show FEM TICS27’s performance across years. We added Table 6 to present FEM TICS27’s performance for 2, 4, 6, 8 and 10 years in predicting dementia or dead with dementia, and refer to this table in the text:

*To demonstrate FEM TICS27’s performance across years, Table 6 shows AUROC for predicting the main outcome, dementia or dead with dementia, in 2, 4, 6, 8 and 10 years. As shown, FEM TICS27’s predictive performance is highest for 2-year prediction and decreases as the prediction timeframe increases. The 10-year AUROC for the full sample is still above 0.9.*

*Table 6. Area under the receiver operating characteristics curve (AUROC) for predicting dementia or dead with dementia for 2, 4, 6, 8 and 10 years.*

|  | *2 years (2008)* | *4 years (2010)* | *6 years (2012)* | *8 years (2014)* | *10 years (2016)* |
| --- | --- | --- | --- | --- | --- |
| *Full sample* | *0.995* | *0.955* | *0.941* | *0.920* | *0.904* |
| *Age 65+ in 2006* | *0.993* | *0.946* | *0.929* | *0.902* | *0.883* |
| *Verified MCI in 2006* | *0.961* | *0.812* | *0.783* | *0.740* | *0.720* |

**Response to Reviewer 2**

1. **In this study present FEM TICS27’s model structure, variables, data sources, and conducts validation of its simulation outcomes against observed HRS data. Information about the method was insufficient in this article.**

We agree with the reviewer that the validation strategy as originally presented in Methods section ‘d. Model validation approach’ may have been confusing. We clarified the approach by adding a high-level overview of the validation strategy in this section:

*We validated the FEM TICS27 model at both the population- and individual-levels. At the population-level, we looked at two outcomes, TICS27 distribution comparisons and 10-year changes in a composite measure of both cognition and mortality. At the individual-level, we assessed FEM’s performance in predicting dementia in 2/4/6/8/10 years and significant decline in TICS27 in two years using receiver operating characteristics (ROC) curves.*

We then continue to explain more technical details of our validation process in the paragraphs that follow.

2. **According to the studies in the literature, it has increased performance. However, this study should explain why it differs from other studies.**

We added the following sentence to the Discussion:

*The increased performance of FEM over other models is likely because it utilizes information on individual characteristics and behavior, like smoking, widowhood, and disease history.*

Unrelated to the editor’s or reviewers’ comments, we found we had incorrectly labeled one of the age categories in our original manuscript. Results were presented for both ages 53+ and 70+, but these should have been labeled with ages 53+ and 65+, respectively. The results remain the same. We have corrected the label in the manuscript and tables.
